# Structure and Corrosion Resistance of Fe40Al5Cr0.2TiB Alloy After Casting and After Homogenization Annealing

**DOI:** 10.3390/ma18020308

**Published:** 2025-01-11

**Authors:** Janusz Cebulski, Dorota Pasek, Magdalena Popczyk, Andrzej Swinarew, Jadwiga Gabor

**Affiliations:** 1 Department of Materials Technology, Faculty of Materials Engineering, Silesian University of Technology, Krasińskiego 8, 40-019 Katowice, Poland; 2 Promobil s.c., Kopernika 12, 40-064 Katowice, Poland; dorota.pasek@promobil.pl; 3 Faculty of Science and Technology, University of Silesia in Katowice, 75 Pułku Piechoty 1A, 41-500 Chorzów, Poland; andrzej.swinarew@us.edu.pl (A.S.); jadwiga.gabor@us.edu.pl (J.G.); 4 Institute of Sport Science, The Jerzy Kukuczka Academy of Physical Education, Mikołowska 72A, 40-065 Katowice, Poland

**Keywords:** intermetallic phase, FeAl alloy, corrosion resistance

## Abstract

This article shows the results of research conducted on the corrosion resistance of the FeAl (Fe40Al5Cr0.2TiB) alloy in two variants: the alloy after casting and after homogenization annealing (1000 °C, 93 h). Analysis of the microstructure of these alloys was conducted on the light microscope, and the phase composition was determined by X-ray diffraction. Resistance to electrochemical corrosion was tested in a 5% NaCl solution using the potentiodynamic polarization technique and electrochemical impedance spectroscopy. The surface of alloys after corrosion tests was examined by scanning electron microscopy. Chemical composition tests were conducted using an energy-dispersive X-ray spectrometer. The structure analysis was made with an electron backscatter diffraction detector. Based on the studies, it was found that the corrosion resistance of the FeAl alloy after homogenization annealing was higher than that of the FeAl alloy after casting. This alloy showed a more non-homogeneous and coarse-grained microstructure compared to the alloy after homogenization annealing. The investigation of the surface condition of FeAl alloys after corrosion tests showed the presence of pits, particularly in the case of the alloy after casting.

## 1. Introduction

Steels are among the most critical engineering materials due to their high strength, excellent deformability, and recyclability, making them essential across diverse industries such as automotive, aerospace, marine, railway, construction, and energy sectors. These attributes contribute to their widespread application in various structural and functional components. However, despite their advantageous properties, steels have notable limitations, including high density and susceptibility to corrosion. These drawbacks have driven the pursuit of alternative materials that can maintain the mechanical properties of steel while offering lower density and improved corrosion resistance.

Metal alloys can be produced through a variety of methods, including grinding in ball mills, electrodeposition, and casting [1,2,3,4]. Recent advancements in material engineering have led to the development of knowledge and manufacturing of innovative casting alloys based on the FeAl intermetallic phase.

Advances in materials engineering have led to the development of knowledge and production of innovative casting alloys based on the intermetallic phase, called intermetallics. These materials belong to the so-called intermediate phases. Currently, the most advanced work in the field of metallurgy and property research is carried out in relation to intermetallics from the Ni-Al, Fe-Al, and Ti-Al systems [5]. Less interest in the TiAl system phases results from the fact that most of the alloys used are two-phase alloys that require the addition of components to stabilize the structure, which increases the cost of their production. Single-phase alloys are brittle and unsuitable for industrial use [6,7]. Similarly, in the case of alloys based on the Ni-Al phase, it is required to obtain reinforcing phases in order to obtain optimal alloy properties, which are precipitated at the grain boundaries as a result of heat treatment [8].

These alloys, composed predominantly of ordered intermetallic phases, stand out due to their unique properties that align well with the requirements of high-performance applications [9,10,11,12,13,14,15].

FeAl alloys are particularly noteworthy due to their 40% aluminum content. Alloys based on the intermetallic phase Fe40Al are single-phase in the entire solid-state range, and in addition, in comparison to Ni-Al and Ti-Al alloys, they are characterized by a lower price of input elements [16]. Materials with a structure predominantly composed of ordered intermetallic phases from the Fe-Al system possess properties that make them suitable as structural materials for components operating at elevated temperatures and in aggressive environments, including exposure to multiple oxidants [17,18]. This resistance is largely attributed to the appearance of a passive aluminum oxide layer on the surface [19]. Additionally, FeAl-based alloys exhibit very good mechanical properties in terms of tolerance to abrasive, erosive, and cavitation wear. To enhance their plasticity and resistance to brittle fracture, these alloys undergo plastic deformation processes, resulting in a fine-grained structure. While these materials are particularly advantageous for high-temperature applications, they may also be suitable for use at room temperature [20].

Some alloy materials often exhibit a coarse-grained and inhomogeneous structure, leading to challenges such as brittleness and susceptibility to localized corrosion. This non-homogeneous structure can create micro-galvanic cells that accelerate corrosion processes, especially in aqueous environments. To mitigate these issues, homogenization annealing is employed. The reduction in the active surface after homogenization annealing is the main reason for improving the corrosion resistance of alloys. This process makes the surface of these alloys more uniform. As far as we know, no information is available on the influence of homogenization annealing on the corrosion resistance of FeAl alloy (Fe40Al5Cr0.2TiB). Alloys based on ordered phases from the Fe-Al system, in particular on the FeAl phase, have been studied since the mid-1990s. As part of research, numerous heat resistance tests were performed in the oxidation process, which were the basis for verification in practical conditions based on operational tests. The tests were conducted on elements used in automotive turbochargers in the hot part, including in the variable blade geometry system, over a distance of 60 thousand km. After the tests, certain local areas of electrochemical corrosion were found, which include the effect of exhaust gas condensation after the engine is switched off and the temperature or initial phase of engine operation being reduced. Among the industries in which these alloys can be used are the energy, chemical, petrochemical, and shipbuilding industries. FeAl-phase alloys can also be used for heating elements [5].

So far, the corrosion resistance of the FeAl alloy has been studied in a corrosive environment containing HCl and H_2_SO_4_. In the available literature, there are no studies determining the electrochemical corrosion resistance of these materials in a NaCl solution. The research presented in this paper seeks to investigate the effects of two states, specifically, after casting and homogenization annealing (1000 °C, 93 h), on the corrosion resistance of FeAl alloy.

## 2. Materials and Methods

The test material comprised samples made from the FeAl alloy (Fe40Al5Cr0.2TiB), with its chemical composition detailed in Table 1. The elements used for smelting included ARMCO iron, ARO aluminum, aluminothermic chromium obtained via the Kroll method, and amorphous boron (Table 1). The melts were conducted using a Balzers VSG-2 vacuum induction furnace (Balzers, Lichtenstein). Initially, the primary components, iron and aluminum, were placed in an alundum crucible. The alloying additives were then introduced into the metal bath sequentially, according to their increasing reactivity. The melting process was performed under a vacuum of 10⁻^2^ Pa, achieved with a rotary pump. The metal bath was heated to 1500 °C and subsequently poured into a mold within the furnace atmosphere, where the melting occurred.

The study’s primary objective was to assess the corrosion resistance of the FeAl alloy in a 5% NaCl solution, both in its as-cast state (sample 1) and after homogenizing annealing (sample 2). Due to the fact that the material after casting was brittle and had a coarse-grained structure and significant segregation of the chemical composition, heat treatment was applied. Homogenizing annealing was carried out at a temperature of 1000 °C for 93 h because, based on previous studies, it was shown that these are the parameters at which microsegregation of the chemical composition is removed.

The research program comprised the following steps:

1. Examination of the alloy’s microstructure after casting (sample 1) and following homogenization annealing (sample 2) utilized a light microscope, specifically the Olympus GX51, prior to conducting corrosion tests. The phase analysis of the FeAl alloys before the corrosion test was performed using a JEOL JDX-7S diffractometer (Jeol, Tokyo, Japan) with copper radiation. Phase identification was performed using PCSIWIN software (version 2.0) and the JCPDS card database (ICDD, 2000). Transmission electron microscopy studies of the substructure were performed on a Hitachi HD-2300A microscope (STEM)( Tokyo, Japan). The quantitative characterization of the FeAl alloy microstructure was performed using the quantitative image analysis program MetIlo (version 9.07);
2. Evaluation of the corrosion resistance of the FeAl alloys was carried out in a 5% NaCl solution employing potentiodynamic polarization and electrochemical impedance spectroscopy (EIS). These electrochemical analyses were conducted with the AUTOLAB system (PGSTAT30, Metrohm Autolab B.V., Utrecht, The Netherlands). A saturated calomel electrode (SCE) was used as the reference electrode, while a platinum grid served as the auxiliary electrode. Potentiodynamic curves were registered within a potential range of ±250 mV relative to the open circuit potential at a rate of 1 mV·s⁻¹. EIS studies were conducted at the corrosion potential across a frequency range from 50 kHz to 0.005 Hz, utilizing a sinusoidal wave with a 10 mV amplitude and recording 10 measurement points per frequency decade. All electrochemical tests were conducted at room temperature;
3. The surface condition of the samples after corrosion testing was examined using a Hitachi S-4200 scanning electron microscope (SEM). The chemical composition was analyzed using an energy-dispersive X-ray spectrometer (EDS) by ThermoNoran (System Seven) (Madison, WI, USA), operated at an electron beam accelerating voltage of 15 keV. The spectrometer is combined with the microscope, as mentioned above. Backscattered electron diffraction was performed using the EBSD INCA HKL detector Nordlys II (Channel 5)—(Hobro, Denmark), which was equipped with the Hitachi S-3400N microscope. The phase analysis of the FeAl alloys after the corrosion test was performed similarly to the alloys before the corrosion test using a JEOL JDX-7S diffractometer.

## 3. Results and Discussion

### 3.1. Characterization Before Corrosion Tests

**Figure 1:**
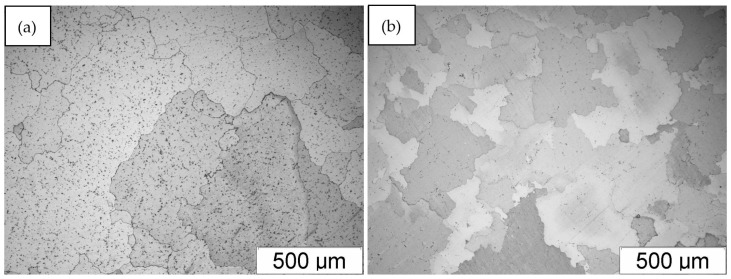
Microstructure of FeAl alloy: (**a**) after casting; (**b**) after homogenization annealing.

The FeAl alloy after casting showed an inhomogeneous and coarse-grained microstructure compared to the more uniform alloy after homogenization annealing (Figure 1). The average surface area of a flat grain cross-section of the FeAl alloy after casting was 0.027 mm^2^, and that of the FeAl alloy after homogenization annealing was 0.022 mm^2^.

**Figure 2:**
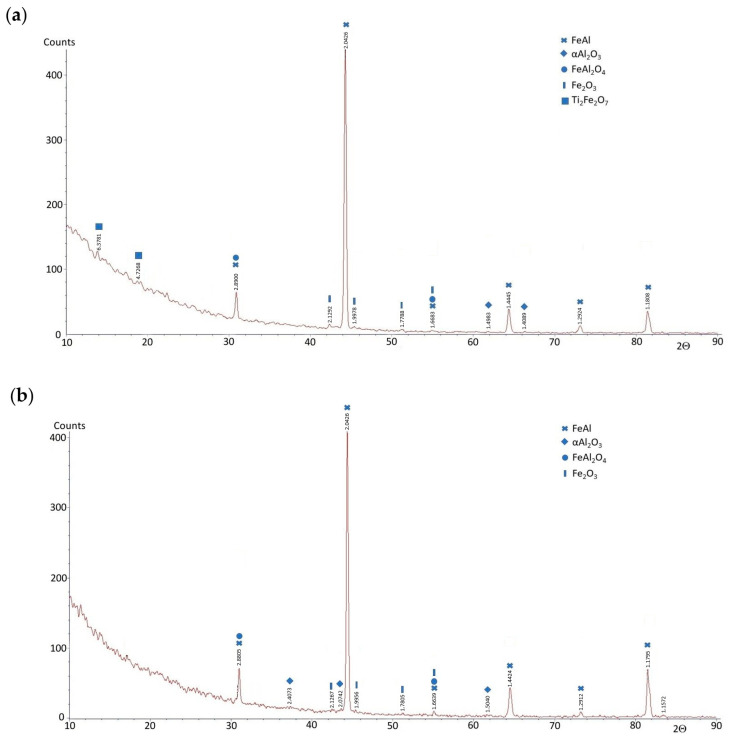
X-ray diffraction patterns for FeAl alloy: (**a**) after casting; (**b**) after homogenization annealing.

**Figure 3:**
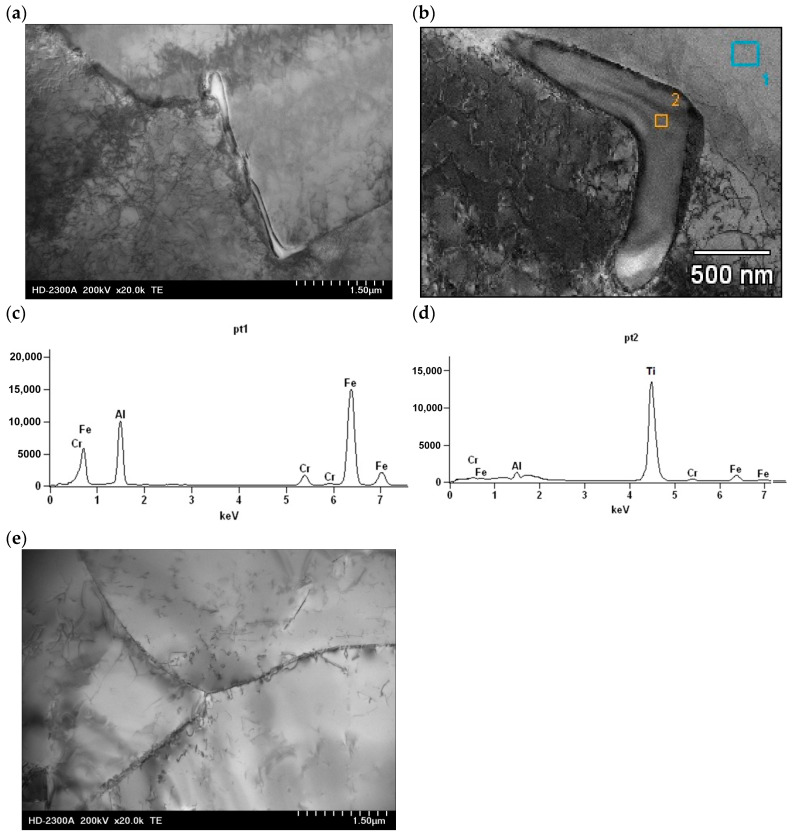
FeAl alloy after casting (**a**,**b**); X-ray spectra of chemical composition with energy dispersion from areas 1 and 2 marked in fig. b (**c**,**d**); FeAl alloy after homogenization annealing (**e**).

To evaluate the phase composition of the FeAl alloy after casting and after homogenization annealing, X-ray diffraction (XRD) analysis was performed in the angular range of 2Θ from 10° to 90°. The phase composition of the FeAl alloy after casting compared to the FeAl alloy after homogenization annealing did not show any significant differences. The XRD analysis of the FeAl alloy after casting showed that this material consists of five phases: FeAl, α-Al_2_O_3_, FeAl_2_O_4_, Fe_2_O_3_, and Ti_2_Fe_2_O_7_ (Figure 2a). The main phase (dominant) of the diffraction pattern is FeAl, while the other phases are present in trace amounts. The phase composition of the FeAl alloy after homogenization annealing differed only slightly from that of the as-cast alloy. The Ti_2_Fe_2_O_7_ phase was not present in the diffraction pattern of the alloy after homogenization annealing (Figure 2b). This may be due to the fact that the chemical composition of the tested alloy contains titanium, which acts as a modifier, improving the plastic properties of the alloy. Studies of the substructure of the FeAl alloy conducted using a transmission electron microscope Hitachi HD-2300A ( Tokyo, Japan) showed that the material after crystallization has numerous defects in the crystal structure distributed in an uneven manner. After crystallization, the material is characterized by a low density of dislocations, and in addition, irregularly shaped precipitates occurred in the forming grains and subgrains (Figure 3a,b). X-ray microanalysis of the chemical composition EDS performed at the place of the precipitate on the subgrain boundary of the FeAl alloy showed that the precipitate is titanium (Figure 3c,d). Heat treatment, especially in alloys based on the intermetallic phase FeAl, causes homogenization of the structure and partial annihilation of precipitates formed in grains and subgrains (Figure 3e).

### 3.2. Corrosion Resistance Tests

**Figure 4:**
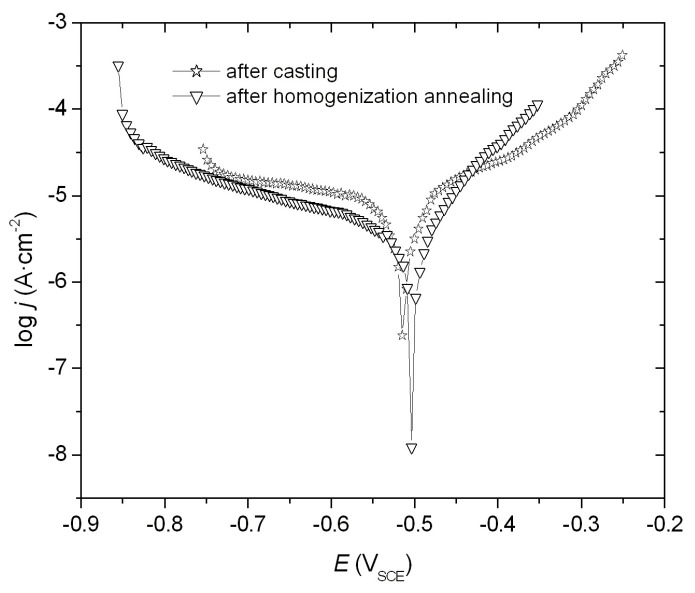
The potentiodynamic curve of log *j* = f(*E*) obtained for the FeAl alloy after casting and FeAl alloy after homogenization annealing (1000 °C, 93 h).

The corrosion resistance of FeAl alloys was evaluated by electrochemical measurements in a 5% NaCl solution. The parameters of corrosion resistance *E*_corr_, *j*_corr_, and *R*_p_ (Table 2) were determined from the obtained log *j* = f(*E*) relationship (Figure 4). The minimum point on the potentiodynamic curve indicates the observed corrosion potential *E*_corr_, which corresponds to the corrosion current density *j*_corr_, directly proportional to the rate of corrosion processes. The results indicate that the FeAl alloy after homogenization annealing exhibits higher corrosion resistance compared to the FeAl alloy after casting. This is demonstrated by a higher polarization resistance *R*_p_, a lower current density, and a shift of the corrosion potential towards positive values (Table 2).

**Figure 5:**
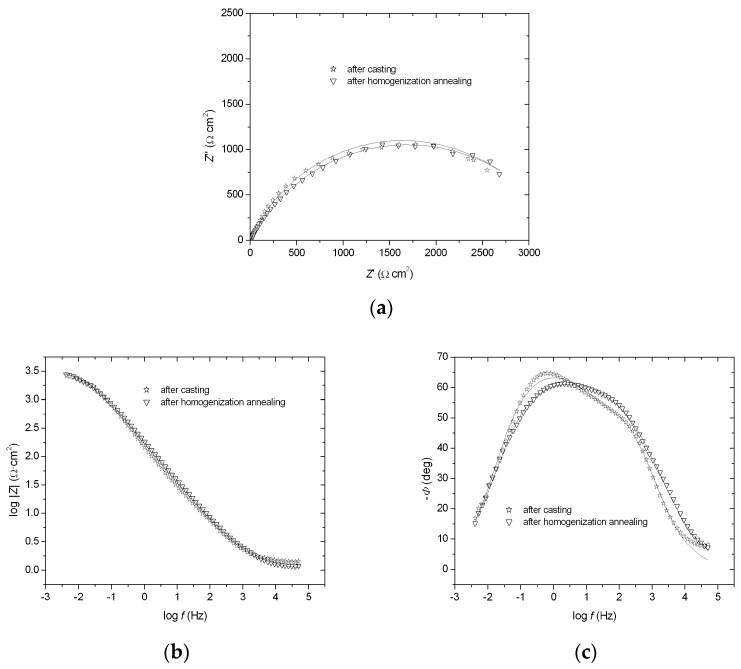
Nyquist and Bode diagrams: −*Z*′ = f (*Z*″) (**a**), log ǀ*Z*ǀ = f (log *f*) (**b**), and −*Φ* = f (log *f*) (**c**) obtained for the FeAl alloy after casting and FeAl alloy after homogenization annealing (1000 °C, 93 h).

The impedance spectra registered at the corrosion potential are presented as Nyquist and Bode diagrams (−*Z*″ = f(*Z*′) and log ǀ*Z*ǀ = f (log *f*), −*Φ* = f (log *f*)) (Figure 5a–c). The EIS measurements showed a semicircle in the whole range of frequencies in complex-plane plots (Figure 5a). To analyze the data, the impedance modulus value was determined at a frequency of 0.1 Hz (Figure 5b). The impedance modulus at low frequencies is often used as a simple indicator for comparing materials [21]. The parameter logǀ*Z*ǀ_0.1_ for the FeAl alloy after homogenization annealing showed a higher value (2.879 Ω·cm^2^) compared to the FeAl alloy after casting (2.850 Ω·cm^2^) (Figure 5b), indicating more effective corrosion resistance after annealing. Moreover, the dependence of −*Φ* = f (log *f*) for the FeAl alloy after homogenization annealing presented a wider range of phase angle independence from the logarithm of frequency compared to the FeAl alloy after casting (Figure 5c). This wider range implies greater corrosion resistance for the annealed alloy. The EIS results were analyzed using a CNLS fitting program (Frequency Response Analysis, Windows version 4.9). It was ascertained that the impedance of FeAl alloys can be described by a CPE2 model (solution resistance, *R*_s_, in series with two parallel *CPE*–*R*_p_ elements) [22]. As a result of the approximation of the experimental data, *R*_p1_,*T*_1_, *ϕ*_1_, *R*_p2_, *T*_2_, *ϕ*_2_, and *R*_s_ parameters were obtained (Table 3), where *R*_1_ and *R*_2_ are the polarization resistances, *T*_1_ and *T*_2_ are the capacity parameters, and *ϕ*_1_ and *ϕ*_2_ are the CPE angles (see [22] and references therein). Also, on the basis of these results, it was found that the FeAl alloy after homogenization annealing is more corrosion-resistant: the value of polarization resistance was higher for this alloy, and the value of capacity parameter was lower, which indicates a smaller interphase surface and therefore improvement in the corrosion resistance of the material (Table 3).

### 3.3. Characterization After Corrosion Tests

**Figure 6:**
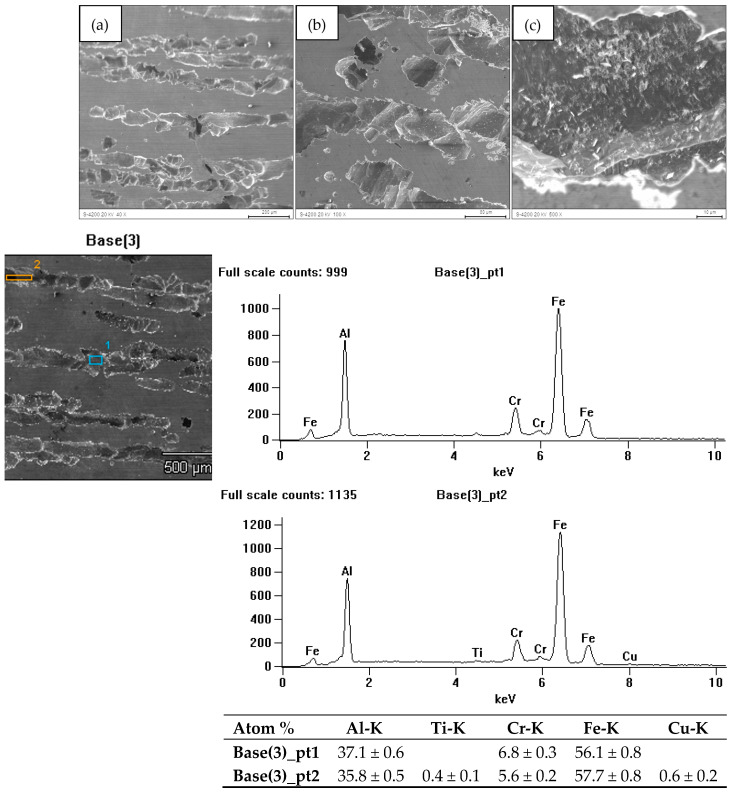
Surface of FeAl alloy after casting and corrosion test (sample 1) (**a**–**c**) along with the X-ray microanalysis of the chemical composition EDS from the areas marked in Fig. Base 3.

**Figure 7:**
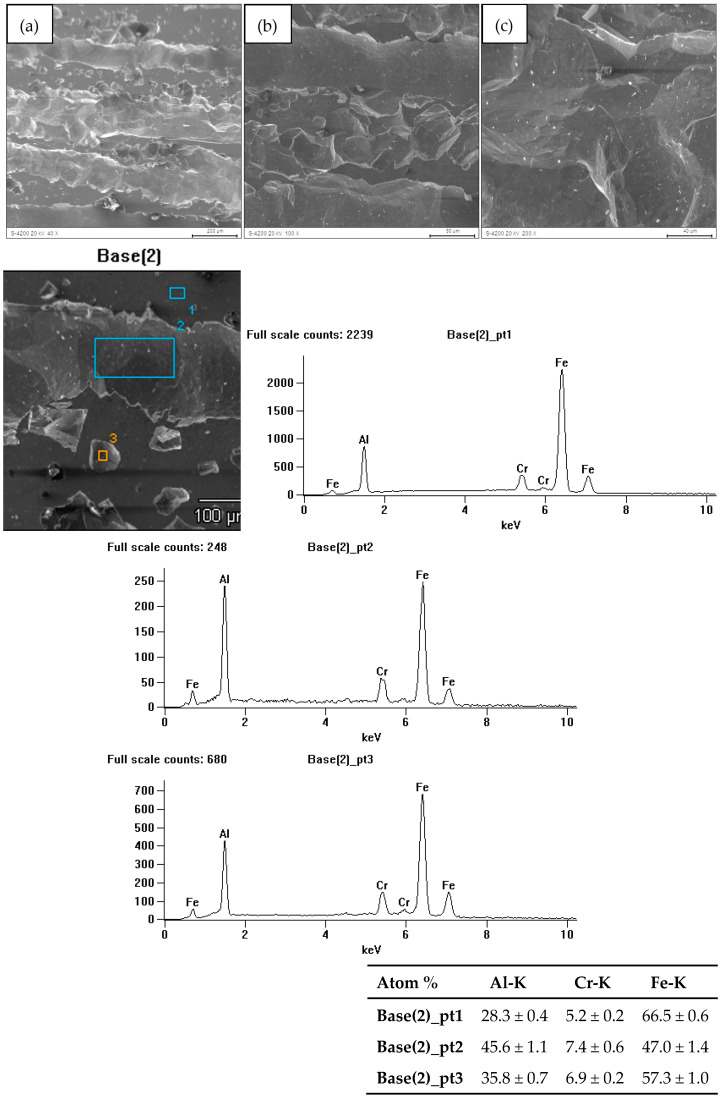
Surface of FeAl alloy after homogenization annealing and corrosion test (sample 2) (**a**–**c**) along with the X-ray microanalysis of the chemical composition EDS from the areas marked in Fig. Base 2.

The research on the surface condition of FeAl alloys after corrosion tests revealed the presence of pits. Their distribution on the surface of the cast material was more non-uniform compared to the material after homogenization annealing. The greatest surface damage, in terms of both the quantity and size of the pits, was found in the cast material. Figure 6 illustrates the surface of the FeAl alloy after casting (sample 1), showing significant surface dissolution and pits running along the grain boundaries. These pits penetrated the material. Figure 7 presents the surface of the FeAl alloy after homogenization annealing (sample 2), showing homogeneous corrosion effects. Additionally, the pits on the annealed material’s surface appeared as gutters. The studies carried out showed that pitting develops primarily inwards, which may result in the loss of whole grains.

Microanalysis of the chemical composition of the surface of the samples after corrosion tests showed a loss of aluminum compared to the initial composition. Moreover, the corrosion tests on the alloy surface revealed only the presence of elements that were part of the tested material.

The course of corrosion processes on the tested samples was not uniform. Some areas were not damaged. As a result of electrochemical processes occurring in microcells on the surface of the sample exposed to the electrolyte, corrosion occurred. Analysis of the surface condition of the material after corrosion tests allows us to assume that grains with a specific crystallographic orientation may be subject to corrosion. Microcells may even be grains of the same phase with different crystallographic orientation. Confirmation of this phenomenon requires further research.

**Figure 8:**
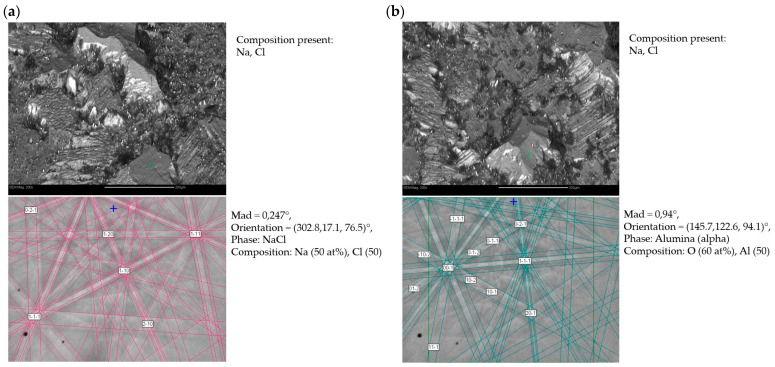
(**a**,**b**) Phase composition results of FeAl alloy after casting and corrosion test (sample 1). Studies conducted in different areas of the sample.

**Figure 9:**
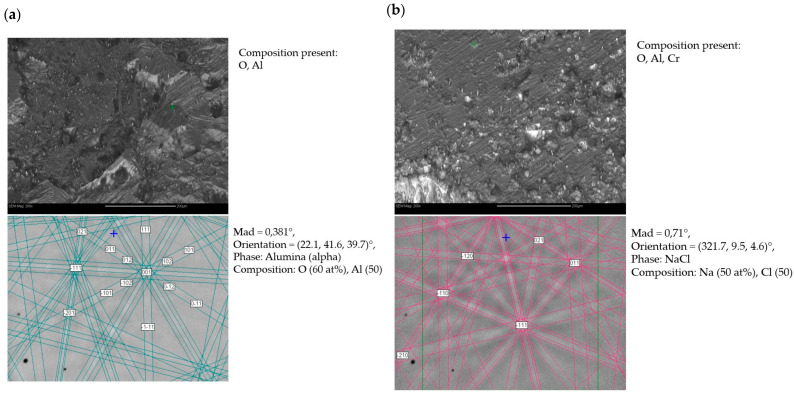
(**a**,**b**) Phase composition results of FeAl alloy after homogenization annealing and corrosion test (sample 2). Studies conducted in different areas of the sample.

**Figure 10:**
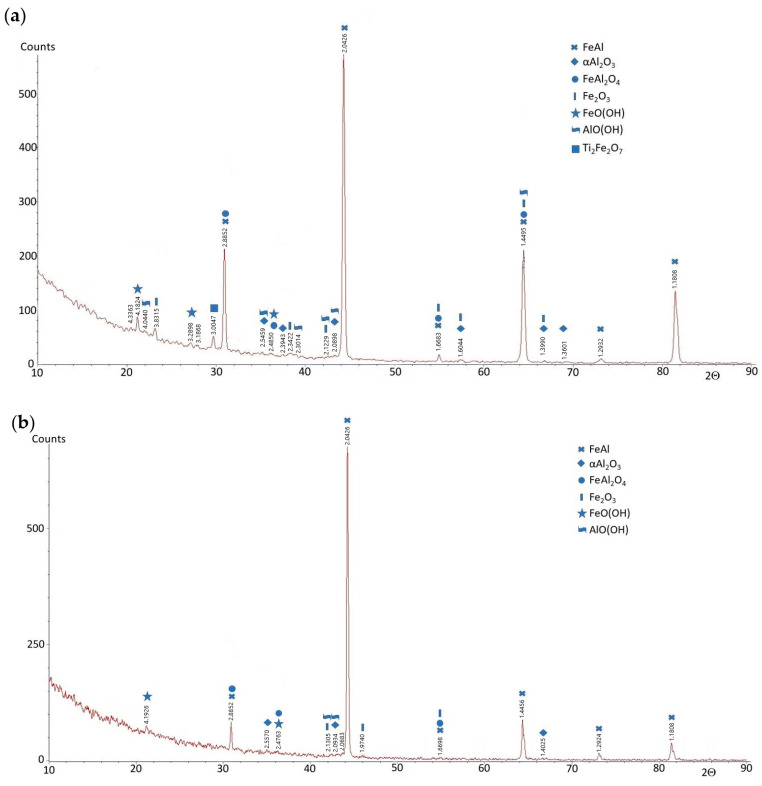
X-ray diffraction patterns for FeAl alloy: (**a**) after casting and corrosion test; (**b**) after homogenization annealing and corrosion test.

Phase composition studies using the EBSD method were performed in two different places (a and b) for sample 1—after casting and corrosion test (Figure 8)—and also in two different places (a and b) for sample 2—after homogenization annealing and corrosion test (Figure 9). These studies showed that in both the as-cast and homogenizing annealed alloys subjected to corrosion tests in NaCl solution, oxide phases of α-Al_2_O_3_ were present. Additionally, there were also NaCl residues, but chlorine did not create the metal chlorides that were part of the tested material. The difference in the case of corrosion processes occurring during the test was a different surface topography; i.e., the alloy after casting subjected to corrosion processes had larger corrosion pits than the alloy after homogenization annealing. Also, previous studies of the FeAl alloy showed that the only stable phase that forms on the surface is α-Al_2_O_3_. This is particularly visible in the case of high-temperature oxidation of this alloy, although in such a case, thermally activated diffusion phenomena play an important role. In this study, initial phase composition analyses were conducted using electron backscatter diffraction (EBSD) (Hobro, Denmark). While EBSD provides valuable insights into the crystallographic orientation and microstructural characteristics of the alloy, it has limitations in confirming phase composition, especially when dealing with complex intermetallic phases. Therefore, X-ray diffraction (XRD) was used to improve the accuracy of phase identification. XRD analysis after the corrosion test was performed in the angular range of 2Θ from 10° to 90°, similar to that of the alloys before the corrosion test. This analysis showed that the phase composition of the FeAl alloys was almost the same as before the test. The FeAl intermetallic phase was still dominant. In the diffraction patterns of these alloys (after casting and after homogenizing annealing), two additional phases appeared: AlO(OH) (aluminum oxyhydroxide) and FeO(OH) (iron oxyhydroxide), i.e., chemical compounds of aluminum/iron, oxygen, and hydrogen, which are products of the oxidation of these metals in an aqueous solution of sodium chloride (Figure 10).

## 4. Conclusions

The X-ray diffraction analysis performed before the corrosion test showed that the phase composition of the FeAl alloys after casting and after homogenization annealing was almost the same, which probably means that the phase composition, in this case, has no influence on corrosion resistance. However, the FeAl alloy after homogenization annealing showed a more uniform microstructure compared to the non-homogeneous and coarse-grained alloy after casting. This inhomogeneous structure can create micro-galvanic cells that accelerate corrosion processes. This was confirmed by corrosion resistance tests in 5% NaCl solution, performed using the potentiodynamic polarization method and electrochemical impedance spectroscopy. It was shown that the FeAl alloy after homogenization annealing (1000 °C, 93 h) is characterized by more favorable corrosion parameters compared to the FeAl alloy after casting. The surface condition of FeAl alloys after casting and after homogenization annealing, subjected to corrosion tests in NaCl solution, indicated their clear differences. The examination of the FeAl alloy surface condition after corrosion tests revealed the presence of numerous pits, especially in the case of the alloy after casting. Analysis of the test results indicated the alloy’s tendency to activate the surface, which should be considered as an unfavorable property of the tested material.

## Figures and Tables

**Table 1:** Chemical composition of FeAl alloy after casting (Fe40Al5Cr0.2TiB alloy).

Compound	Al	Cr	Ti	B	Fe
% mas.	24.53	5.80	0.19	0.01	rest
% at.	40.10	4.86	0.18	0.06	rest

**Table 2:** Corrosion resistance parameters of FeAl alloys.

FeAl Alloys	*E*_corr_ [V]	*j*_corr_ [μA·cm^−2^]	*R*_p_ [Ω·cm^2^]
After casting	−0.513	5.8	4463
After homogenization annealing	−0.503	3.8	7134

**Table 3:** EIS parameters determined for FeAl alloy.

FeAl Alloys	*R*_p1_ (Ω·cm^2^)	*T*_1_	*ϕ*_1_	*R*_p2_ (Ω·cm^2^)	*T_2_*	*ϕ*_2_	*R*_s_ (Ω·cm^2^)
After casting	1.78	0.000347	0.65	3270	0.000206	0.75	1.05
After homogenization annealing	1.96	0.000335	0.47	3390	0.000097	0.70	1.03

## Data Availability

The original contributions presented in this study are included in the article. Further inquiries can be directed to the corresponding authors.
